# Rethinking Histones as Negative Markers for the Characterization of Extracellular Vesicles

**DOI:** 10.1002/jex2.70149

**Published:** 2026-05-15

**Authors:** Javaria Munir

**Affiliations:** ^1^ Department of Biology William and Mary Williamsburg Virginia USA

**Keywords:** characterization, extracellular vesicles, histones, markers

## Introduction

Extracellular vesicles (EVs) are membrane‐bound vesicles that function as mediators of cell‐cell communication in unicellular eukaryotes (such as yeast), insects (such as *Drosophila)*, simple invertebrates (such as *C. elegans*), lower vertebrates (such as zebrafish) and higher vertebrates (such as mammals) Lötvall et al. 2014, Mulzer et al. 2025, Szczepańska et al. 2024, Sanchez‐Lopez et al. 2022, Xu et al. 2025. Although the term “exosomes” is often used interchangeably with EVs, exosomes specifically originate from the endocytic pathway through multivesicular bodies (MVBs). This distinguishes them from ectosomes (commonly referred to as microvesicles), which bud directly from the plasma membrane of living cells, and apoptotic bodies, which are released from the plasma membrane during cell death Kalluri and LeBleu 2020. To reduce ambiguity, minimal information for studies of extracellular vesicles (MISEV) 2023 recommends the use of operational terms and emphasizes explicit reporting of size ranges rather than strict size‐based definitions and cautions against labeling vesicles as ‘exosomes’ without direct evidence of endocytic biogenesis Welsh et al. 2024. In practice, EV characterization relies mainly on protein markers that provide indirect evidence of origin rather than definitive proof. Histones, which are abundant nuclear proteins involved in chromatin organization, are frequently released during apoptosis, necrosis, or other forms of cell death and may therefore appear in extracellular preparations as contaminants. At the same time, several studies have reported histone‐associated signals in EV fractions, raising questions about whether these proteins represent contamination, methodological artifacts, or biologically meaningful cargo. Here, I examine how histone presence or depletion has been interpreted in EV studies and propose a framework to distinguish contamination from functional association.

MISEV2018 and MISEV2023 organize EV‐associated proteins into five marker groups Welsh et al. 2024, Théry et al. 2018. Three are strongly recommended: (i) transmembrane or GPI‐anchored proteins, (ii) cytosolic proteins, and (iii) proteins associated with non‐EV co‐isolated structures. Two optional groups provide contextual information: (iv) proteins from organelles other than the plasma membrane or endosomes, and (v) secreted proteins that may form a protein corona around EVs Welsh et al. 2024, Théry et al. 2018. Conceptually, this framework encourages contextual interpretation of markers rather than rigid inclusion or exclusion. Despite this shift in framework, many studies still follow MISEV2014‐era practice, which emphasizes a set of positive markers alongside a set of “intracellular proteins” associated with non‐plasma membrane and non‐endosomal compartments and may indicate absence or under‐representation of EVs to control for contamination Lötvall et al. 2014, Yang et al. 2023, Shuwari et al. 2024. Histones are widely used in this latter category as indicators of nuclear contamination Yang et al. 2023, Shuwari et al. 2024, Kapoor et al. 2023, Dickman et al. 2017. Importantly, this perspective does not suggest that current guidelines advocate the use of histones as strict exclusion markers, but rather that their interpretation in the literature has at times been simplified.

The presence of nuclear proteins, including histones, in EV preparations has raised legitimate concerns regarding contamination from apoptotic bodies or residual cellular debris. Methodological studies have demonstrated that histone enrichment in EV fractions frequently correlates with apoptotic vesicles and insufficient pre‐clearing or lack of density‐based separation, rather than bona fide small EVs Tauro et al. 2012, Coumans et al. 2017. Importantly, optimized isolation strategies incorporating sequential centrifugation and density gradient purification substantially reduce nuclear contamination and markedly diminish histone‐associated material in EV preparations Lobb et al. 2015. Table 1 summarizes representative studies reporting histones as contamination indicators and as EV‐associated components across different experimental contexts

**TABLE 1:** Summary of representative studies highlighting methodological evidence of histone contamination under specific biological contexts versus histone as legitimate EV components.

Category	EV Source / Model	Isolation
Histones as contaminants	Malignant epithelial cancer cells, bone marrow mesenchymal stem/stromal cells, foreskin fibroblasts and non‐malignant epithelial cells Kapoor et al. 2024.	Size‐exclusion liquid chromatography
	Colorectal cancer cells Liu et al. 2022	UC^1^
	Bronchial epithelial cells Benedikter et al. 2017	Ultrafiltration + Size‐exclusion chromatography (SEC)
	Plasma Bettio et al. 2023	UC + ExoEasy kit (Qiagen)
	Plasma Gelibter et al. 2022	Size‐exclusion chromatography
Histones as functional EV components	Breast cancer cells Nangami et al. 2014	—
	Brain glial cells Singh et al. 2025	UC + Density gradient centrifugation
	Bladder cancer cells Huang et al. 2025	UC + SEC
	Patient urine / prostate cancer cells Pang et al. 2024	SEC
	Bone marrow derived macrophages Nair et al. 2018	Density gradient centrifugation

^1^ Ultracentrifugation.

The interpretation of histones solely as indicators of nuclear contamination warrants reconsideration in light of emerging evidence. While histone detection has been attributed to apoptotic bodies or nonspecific adsorption during high‐speed centrifugation, accumulating evidence indicates that histones can associate with sEVs in regulated, biologically meaningful ways (see Table 1) Jeppesen et al. 2019, Théry et al. 2001. Importantly, not all histones behave equivalently extracellularly: core histones, particularly H2B and H3, have been reported as membrane‐associated components of EVs under stress conditions such as hypoxia, LPS (lipopolysaccharides), heat, oxidative stress. However, the linker histone H1 showed variable trend regarding their secretion as associated part of EVs. However, for histone H1, it remains unclear whether it is integral part of the EV membrane or loosely associated as assessed by trypsin protection assays Singh et al. 2025. The experimental evidence of histone secretion associated with EV membrane relies on cell lines under stress conditions consistent with the findings of increased extracellular histone secretion in biofluids in diseased state Singh et al. 2025. However, whether this trend is also applicable to healthy and normal conditions is an open question. Overall, depending on cell type, histone variant, and context, histones may or may not associate with the EV surface or be present within EVs.

Hence, multiple independent studies report that histone–EV association is not solely an artifact of contamination and may bind EV membranes independently of DNA and nucleosome formation, consistent with a cytosolic rather than chromatin‐derived origin Singh et al. 2025. Incorporation of histones in EVs, whether as membrane‐associated components or cargo may represent an evolutionarily conserved phenomenon, suggesting the functional importance of histones in EVs Baquero et al. 2025. In infection models, such as rotavirus‐infected intestinal epithelial cells, histones are detected in both EVs and apoptotic bodies and modulate immune responses, underscoring context‐dependent physiological relevance Bautista et al. 2015, Schiera et al. 2016. Selective packaging further supports regulation: in melanoma cells, the linker histone H1.0 and its mRNA are preferentially loaded into EVs *via* RNA‐binding proteins, indicating active sorting rather than passive leakage Schiera et al. 2016.

Functional consequences of EV‐associated histones further strengthen this argument. EV‐associated histones can influence recipient cells through defined mechanisms. For example, sulforaphane‐treated fibroblast‐derived EVs deliver the endogenous HDAC1 inhibitor “maspin” in recipient cardiomyocytes and facilitate cardioprotection Papini et al. 2023. In experimental systems, circulatory DNA associated with the EV corona *via* histones (and other proteins) can transfer drug resistance, alter transporter expression, and modulate metabolic pathways Tutanov 2022. Clinical studies extend these findings in vivo, where histones and DNA complexes on the EV corona reflect donor cell states and provide diagnostic or prognostic value in cancer, cerebrovascular disease, sepsis, heat stroke, and viral infection Tutanov 2022. Notably, disease‐specific enrichment, receptor‐mediated signaling, and strong correlations with clinical outcomes are difficult to reconcile with a model of random contamination.

Additional evidence demonstrates that nuclear‐derived molecules are bona fide EV cargo rather than mere contaminants. In A549 cells, chromatin DNA associated with the transcription factor FOXM1 is selectively loaded into cytoplasmic LC3–FOXM1–DNA vesicles during autophagy and secreted *via* EVs, as confirmed by immunoblotting and qPCR Feng 2024, Seo et al. 2022, Zhang et al. 2024 Unlike DNA‐independent histone associations, this process demonstrates active, regulated packaging of nuclear molecules. Importantly, FOXM1‐chromatin DNA fragments can be delivered to recipient cells *via* EVs and expressed when they carry functional genes, providing a clear example of how transcription factors can specify nuclear‐derived DNA for EV‐mediated intercellular transfer Zhang et al. 2024. Together, these observations reinforce that nuclear components, whether histones or chromatin DNA, are purposeful EV cargo capable of modulating recipient cell function. Also, although mechanistically distinct from DNA‐independent histone association, these observations converge on a common conclusion: nuclear origin alone does not imply contamination.

EV‐associated histones also mediate functional interactions with recipient cells. Uptake studies show that histones on EVs enhance internalization *via* syndecan‐4, with fetuin‐A facilitating adhesion through heparan sulfate proteoglycans Nangami et al. 2014, Ochieng et al. 2018. In disease contexts, histones contribute to functional outcomes: EVs from gemcitabine‐resistant bladder cancer cells transfer resistance to recipient cells, with histone H3.2 (H3C14) enriched in EVs and modulating nucleoside transporter expression and metabolic enzymes Huang et al. 2025, Balbinotti et al. 2020, Yokoi et al. 2019. In prostate cancer, elevated H4 in patient urine EVs demonstrates diagnostic potential, outperforming PSA and enabling improved risk stratification Nair et al. 2018. Plasma EVs containing H3 and other proteins also serve as biomarkers for cognitive impairment in cerebrovascular disease Feng 2024.

These findings, together with the cancer and metabolic studies described above, demonstrate that histone‐associated EVs play functional roles across diverse pathological contexts. Histones in EVs also have direct clinical and physiological relevance. For instance, in COVID‐19 plasma, extracellular histones and procoagulant EVs independently promote thrombosis, while in heat stroke, EV‐associated histone H3 levels correlate strongly with organ dysfunction and disease severity, distinguishing survivors from non‐survivors with high sensitivity and specificity (AUC ≈ 0.97) Li 2021, Eustes et al. 2024. These observations further underscore that histones are regulated, functional EV cargo rather than mere contaminants.

Taken together, the evidence indicates that histones associated with EVs can be regulated, functional, and clinically informative. If histones were merely contaminants, one would not expect selective loading, conserved mechanisms, receptor‐mediated uptake, or reproducible disease‐specific enrichment. These findings support a more nuanced, context‐dependent interpretation of histones in EV studies. Accordingly, studies should report cell source, condition of cell culture, isolation strategy, specific histone variant, functional validation, post‐translational modification, integral association with EV‐membrane or a part of EV cargo proteome, DNA associated histones or DNA free histones, monomer or multimer and proper controls to rule out contamination. Adopting such an approach is essential to avoid mischaracterizing EV preparations in the context of histones and to fully capture EV‐mediated biology in health and disease.

## Conflicts of Interest

The author declares no conflicts of interest.

## Data Availability

Data sharing not applicable to this article as no datasets were generated or analysed during the current study.
